# Inferior vena cava distensibility index predicting fluid responsiveness in ventilated patients

**DOI:** 10.1186/2197-425X-3-S1-A600

**Published:** 2015-10-01

**Authors:** J Lujan Varas, C Martinez Díaz, R Blancas, O Martinez Gonzalez, B Llorente Ruiz, R Molina Montero, C Arenillas Juanas, A Pardo, L Alcázar Sánchez-Elvira, JA Cambronero Galache

**Affiliations:** Hospital Principe de Asturias, Madrid, Spain

## Introduction

Echocardiography is a non-invasive procedure which enables full assesment of cardiac function. The inferior vena cava (IVC) is a compliant blood vessel, easily distended, especially in cases of hypovolemia. Assessment of the physiologic characteristics of the IVC provides a rapid distinction between low and high volume states and offers the clinician a rapid, noninvasive way to guide resuscitation in critically ill patients.

## Objectives

To assess the reliability of the distensibility of inferior vena cava (dIVC),measured by ultrasound, as an indicator of fluid responsiveness in ventilated patients

## Methods

Observational prospective study in a 14-bed Intensive Care Unit . We enrolled 15 patients requiring advanced hemodynamic monitoring (PiCCO, Vigileo, Swan-Ganz catheter). The dIVC was calculate as (maximum diameter - minimum diameter)/minimum diameter and possible responders were defined as dIVC >18%.IVC assessment was done just proximal to the hepatic veins, which lie approximately 0.5 to 3 cm from the right atrium, following the American Society of Echocardiography guidelines. Hemodynamic parameters were collected at baseline and after a fluid challenge. Fluid challenge was made by de maneuver of passive legs raising (PLR) that mimics a fluid challenge of 300 ml. Fluid responsiveness was defined by an increase of > 15% in cardiac output. Demographics characteristics, reason of ICU admission, severity of illness by APACHE and necessity of vasopressor support were also collected.

## Results

We included 15 patients with an age mean of 64.67 + 14.1, 40% male. Abdominal septic shock was the most frequent reason of ICU admission(40%),respiratory (20%),cardiogenic (13.3%) and others(20%).Median APACHE was 19.27 + 5.86. All patients were on mechanical ventilation with PEEP mean 11.40 + 3.74. All patients were on sensual rhythm and 80% needed vasopressor support. Advance hemodynamic monitoring was made by using PiCCO, Vigileo and Swan-Ganz catheter, 60%, 26.7% and 13.3%, respectively. dIVC was > 18% in 4 patients (26.7%) and 2 patients (50%) responded to fluid challenge. In 11 patients (73.11%) dIVC was < 18% and 10 of them (90.9%) didn’t respond to fluid challenge. Statistical analysis showed no significant differences (p > 0.05).

## Conclusions

Assessment of the IVC distensibility index in mechanically ventilated patients provides a useful and reliable tool in predicting response to volume in critically ill patients. Although our data do not show a statistical significance probably due to sample size, measuring the VCI should be part of a hemodynamic assessment specific protocol to evaluate the necessity or not of volume, that it is so important in the evolution of critical patients.

## References

[1] Barbier C, Loubieres Y, Schmit C, Hayon J, Ricome JL, Jardin F, Vieillard-Baron A (2004). Respiratory changes in inferior vena cava diameter are helpful in predicting fluid responsiveness in ventilated septic patients. Intensive Care Med.

